# Changes in *Brassica oleracea* Leaves Infected With *Xanthomonas campestris* pv. *campestris* by Proteomics Analysis

**DOI:** 10.3389/fpls.2021.781984

**Published:** 2022-02-08

**Authors:** María Tortosa, Pablo Velasco, Víctor Manuel Rodríguez, María Elena Cartea

**Affiliations:** Group of Genetics, Breeding and Biochemistry of Brassicas, Misión Biológica de Galicia, Spanish Council for Scientific Research (CSIC), Pontevedra, Spain

**Keywords:** proteomics, biotic stress, iTRAQ, mass spectrometry, *Brassica oleracea*, *Xanthomonas campestris*

## Abstract

Understanding plant’s response mechanisms against pathogenesis is fundamental for the development of resistant crop varieties and more productive agriculture. In this regard, “omic” approaches are heralded as valuable technologies. In this work, combining isobaric tags for relative and absolute quantification (iTRAQ) technology with mass spectrometry, the proteomes from leaves of *Brassica oleracea* plants infected with *Xanthomonas campestris* pv. *campestris* (Xcc), and control plants at two different post-infection times were compared. Stronger proteomic changes were obtained at 12 days post-infection in comparison with 3 days. The responses observed involved different cell processes, from primary metabolism, such as photosynthesis or photorespiration, to other complex processes such as redox homeostasis, hormone signaling, or defense mechanisms. Most of the proteins decreased in the earlier response were involved in energetic metabolism, whereas later response was characterized by a recovery of primary metabolism. Furthermore, our results indicated that proteolysis machinery and reactive oxygen species (ROS) homeostasis could be key processes during this plant–pathogen interaction. Current data provide new insights into molecular mechanisms that may be involved in defense responses of *B. oleracea* to Xcc.

## Introduction

During their vital cycle, plants are exposed to several unfavorable growing conditions, which often cause significant damages or even plant death. Among these conditions, pathogen attack is one of the most destructive stresses plants have to cope with.

Productivity and quality of crops are seriously affected by numerous diseases caused by bacteria, which pathovars belonging to the *Xanthomonas campestris* species occupy an outstanding place due to their economic impact (Mansfield et al., 2012). One of the most notable of these pathovars is *Xanthomonas campestris* pv. *campestris* (Pammel) Dowson (Xcc), the causal agent of black rot of crucifers that affects all cultivated brassicas. The symptoms of black rot include the characteristic V-shaped chlorotic lesions originating from the leaf margin and blackening of the veins (Williams, 2007).

Crops from *Brassica* genus are among the 10 most economically important vegetables in the global agriculture and markets (Cartea et al., 2011). This genus includes a variety of important crops belonging to *Brassica oleracea* species, such as broccoli, cabbage, cauliflower, kale, and Brussels sprouts. Due to the Xcc infection, the global productivity of these crops has always been below its optimal capacity (Lema et al., 2012). Despite different studies focused developing *B. oleracea* black rot-resistant cultivars, to the authors’ knowledge, the use of resistant cultivars has only had limited success, and available sources with useful are scarce (Vicente and Holub, 2013). Thus, it is necessary to focus our efforts on elucidating the molecular responses produced in the plant during the pathogenesis to understand better how to develop control measures against Xcc.

The molecular responses produced in the plant under any stress include developmental and physiological alterations, which generate important changes on the genome, proteome, and metabolome. Since proteins are key regulators of cellular processes, investigations into proteome alterations can provide important information on how plants cope with stress (Ghosh et al., 2017). In this regard, “omic” approaches are heralded as valuable technologies for monitoring the biological status of an organism and are being applied in plant science to identify key biomolecules involved in plant stress response. However, changes in transcription often do not correspond to changes in protein expression and thus, a comparative proteomic analysis would be an efficient and powerful approach to screen expressed proteins in relation to plant–pathogen interactions. Proteomics has been successfully employed to better understand defense mechanisms in different plant–pathogen systems. Proteomic and bioinformatic approaches specifically are increasingly being applied to address biochemical and physiological effects in response to biotic stresses in plants (Hu et al., 2015; Liu et al., 2019). To understand plant defense systems, it is crucial to identify the complex signaling cascades and the multiple biochemical pathways activated by the pathogen. Proteomic approach allows monitoring differences in abundance of proteins present at sampling and allows studying the changes implied in the plant–pathogen interactions (Ahmad et al., 2016).

To date, different proteomic studies have been performed to analyze the Xcc–*Brassica* interaction (Andrade et al., 2008; Villeth et al., 2009; Akimoto et al., 2014; Santos et al., 2019; Islam et al., 2021). These works allowed us to understand essential processes related to the disease development and the identification of several factors responsible of the Xcc virulence, such as the type III secretion system (White et al., 2009) and indicate that induction of H_2_O_2_ as oxidative stress and proteolysis-related protein accumulation occurred in susceptible interactions (Islam et al., 2021). Some proteomic studies with *Brassica* as a key actor during the Xcc pathogenesis, as the work performed by Villeth et al. (2016), used the classical 2D gel-based approach, which has many limitations, such as problems with reproducibility and quantification or low abundance proteins obscuration (Fuller and Morris, 2012; Santos et al., 2019). Recently, proteomic data of *B. oleracea*–Xcc interaction have been reported in several studies (Santos et al., 2017, 2019; Ribeiro et al., 2018; Zhang et al., 2020; Islam et al., 2021) by using different approach as label-free shotgun 2D-nanoUPLC/MS*^E^* and liquid chromatography–tandem mass spectrometry (LC–MS/MS) (Santos et al., 2017; Islam et al., 2021). Technologies, such as isobaric tags for relative and absolute quantification (iTRAQ), have been developed to overcome the problems derived from gel-based approaches. This labeling method makes possible to identify and quantify proteins from up to 8 different samples within the same experiment, removing reproducibility and quantification limitations.

In this study, we carried out a proteomic approach by combining iTRAQ labeling, LC separation, and MS approach to investigate the protein changes occurring in the complete set of proteins—the “proteome”—of *B. oleracea* plants infected with Xcc race 1 at different time infection points.

## Materials and Methods

### Plant Material

The doubled haploid broccoli line “Early Big” (*B. oleracea* var. *italica*), the parental population mapping BolTBDH, was used in this study. Plants were sown in plastic pots containing *Sphagnum* peat (Gramoflor GmbH & Co., Vechta, Germany) in a greenhouse with a minimum temperature of 20°C during the day and 15°C during the night, venting at 25°C and 60% of humidity.

### Inoculation With *Xanthomonas campestris pv. campestris*

**FIGURE 1:**
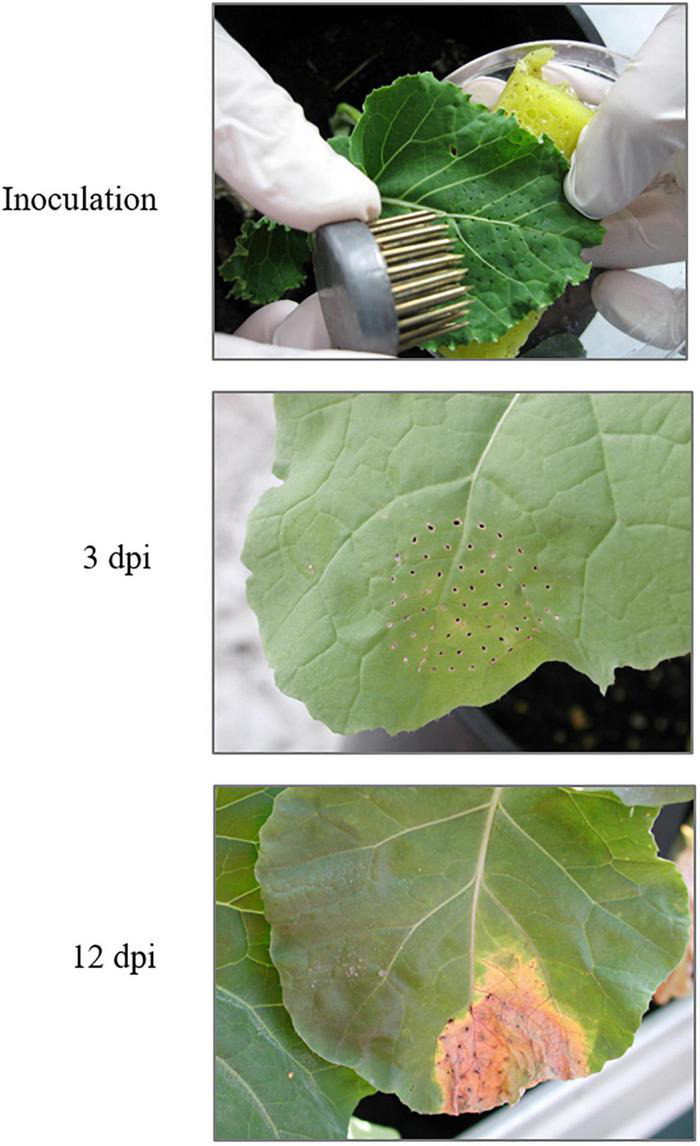
Inoculation and symptoms in *Brassica oleracea* leaves caused by *Xanthomonas campestris* pv. *campestris* 3 dpi and 12 dpi. dpi: days post-infection (source: adapted from Tortosa et al., 2019).

The Xcc race 1 strain HRI3811 was provided by Warwick HRI (Wellesbourne, United Kingdom). Race 1, along with race 4, is the most virulent among 9 described races. Bacterial cultures were grown in potato dextrose agar (PDA) plates (Sigma-Aldrich, Inc. Madrid, Spain) at 30°C in a rotary incubator at 100 rpm for 48 h. Then, the bacterial culture was detached from the plate and diluted in sterile water. Turbidity of the suspension was measured with a spectrophotometer Beckman Coulter DU 62 (Brea, CA, United States) at a wavelength of 600 nm, and the suspension was diluted to reach an absorbance of 0.5, which corresponds with a concentration of 5 × 108 cfu/ml. Plants at six leaf stage were inoculated at the third leaf from the youngest one of each plant was inoculated by using the multiple needles method according to Lema et al. (2007) (Figure 1). Control plants were mock-inoculated following the same procedure to remove the effect of the mechanical damage in the plant response. Four biological replicates per each condition (16 plants) were collected at 3 and 12 -days post-infection (dpi) from control and infected plants. These infection time-points were selected according to the symptom appearance; 3 dpi was taken as symptom starting point and around 12 dpi plants already exhibit cell death at the infection site followed by spreading chlorosis and secondary necrosis in the surrounding uninfected tissue (Figure 1).

### Protein Extraction

For protein extraction, whole fresh leaves were homogenized in ice with a mortar during 10 min using 18 ml of extraction buffer [Tris-HCl 50 mM, ethylenediaminetetraacetic acid (EDTA) 1 mM, and KCl 1 M, pH 7.5] and 50 mg/g of leaf of polyvinylpolypyrrolidone (PVPP). The resulting solutions were centrifuged at 18,000 × *g* for 40 min at 4°C and filtered with cloth filters to remove any vegetal debris. Protein quantification was carried out by using both Bradford and Nanodrop A280 methods.

### Processing of Samples for Isobaric Tags for Relative and Absolute Quantification Labeling

Protein extracts obtained from 16 different samples (2 treatments, 2 dpi, and 4 biological replicates) were analyzed independently. About 500 μl of each sample were concentrated to a final volume of 100 μl with Amicon ultrafiltration units (3 kDa MWCO, Millipore, Billerica, MA, United States) and then cleaned up by pure pre-chilled acetone in a volume six times than that of the protein samples to be precipitated overnight at −20°C. Protein pellets were air-dried and then resuspended in 25 μl dissolution buffer (0.5 M triethylammonium bicarbonate, TEAB). Equal amounts of proteins (25 μg) from each sample were reduced, alkylated, and digested with trypsin by following the supplier’s instructions (AB Sciex, Foster City, CA, United States). Then, iTRAQ labeling was performed according to the supplier’s instructions (AB Sciex, Foster City, CA, United States). Due to 8 different iTRAQ tags were available, comparative analysis of a set 16 samples was feasible within only two MS run. The samples were labeled as follows: control 1, 113; control 2, 114; control 3, 115; control 4, 116; inoculate 1, 117; inoculate 2, 118; inoculate 3, 119; and inoculate 4, 121. iTRAQ-labeled peptides from the same day of analysis (3 and 12 days) were mixed and desalted using reversed phase columns (Pierce C18 Spin Columns, Thermo Fisher Scientific, Rockford, IL, United States) prior to liquid chromatography coupled to mass spectrometry (LC–MS) analysis.

### Liquid Chromatography Coupled to Mass Spectrometry Analysis

The peptide mixtures were firstly separated by off-line reversed-phase liquid chromatography (RP–LC) at basic pH (pH = 9) to lower its complexity. The separation was performed on a HP 1200 system (Agilent Technologies, Santa Clara, CA, United States) employing a C18 reversed-phase column (Zorbax extend C18, 100 3 2.1 mm id, 3.5 mm, 300 Å; Agilent Technologies, Santa Clara, CA, United States). The flow rate used was 0.2 ml/min, and the mobile phases used were 95:5 water:acetonitrile 10 mM NH_4_OH (mobile phase C) and 90:10 acetonitrile:water 10 mM NH_4_OH (mobile phase D). The LC gradient was the following: 0% D from 0 to 10 min; 0–60% D from 10 to 50 min; 60–100% D from 50 to 52 min; 100% D from 52 to 58 min; 100–0% D from 58 to 60 min; and 0% D from 60 to 80 min.

The chromatogram was produced using an ultraviolet (UV) detector at 214 nm. Several fractions were pooled post-collection (FC203B fraction collector, Gilson, Middleton, WI, United States) based on the peak intensity of the UV trace. Each fraction were dried in a vacuum concentrator (Savant Universal SpeedVacVacuum System UVS400A, Thermo Scientific, Waltham, MA, United States) and dissolved in 0.1% trifluoroacetic acid (TFA) and 2% acetonitrile; 5 ml of this sample was injected into a capillary trap column (0.5 mm × 2 mm, Michrom Bioresources, Auburn, CA, United States) at a flow rate of 15 μl/min. Peptides were desalted for 10 min and loaded onto a C18 column (Integrafit C18, ProteopepTM II, 75 mmid, 10.2 cm, 5 mm, 300 Å; New Objective, Woburn, MA, United States) at a constant flow rate of 350 nl/min to perform the separation. Then peptides were separated using linearly increasing concentration of acetonitrile in buffer B (0.1% TFA and 95% acetonitrile), and eluates were deposited onto an Opti-TOF LC MALDI target plate (1,534-spot format; AB Sciex, Framingham, MA, United States) with a speed of one spot per 15 s using the Sun Collect MALDI Spotter/Micro Collector (SunChrom Wissenschaftliche Geräte GmbH, Friedrichsdorf, Germany). Before spotting, 0.3847 μl of the LC microfractions were mixed with MALDI matrix (3 mg/ml a-cyano-4-hydroxycinnamic acid in 70% acetonitrile and 0.1% TFA containing 10 fmol/μl angiotensin as internal standard). Peptide-containing LC spots were analyzed in a 4800 MALDI-TOF/TOF instrument (AB Sciex, Framingham, MA, United States) with a 200-Hz repetition rate (Nd:YAG laser). MS full-scan spectra were acquired from 800 to 4,000 m/z. A total of 1,500 laser shots were accumulated for each time-of-flight MS spectrum at a fixed laser intensity of 3,000 kV. After the screening of all LC-MALDI sample positions in MS positive reflector mode, the fragmentation of automatically selected precursors was performed to generate fragment ions that provided sequence information for the peptide and reporter ions. Tandem MS mode was operated with 1 kV of collision energy with collision-induced dissociation (CID) gas (air) over a range of 60 to −20 m/z of the precursor mass value. The precursor mass window was 200 ppm (full width at half-maximum) in relative mode. Automated precursor selection was done using a job-wide interpretation method (up to 12 precursors/fraction, signal-to-noise lower threshold 80) with a laser voltage of 4,000 kV and 2,000 shots/spectrum at medium CID collision energy range. A second job-wide precursor selection was done excluding those precursors previously fragmented and using a lower signal-to-noise threshold of 50 to identify peptides coming from low-abundance proteins. Data from both MS/MS acquisitions were used for data processing and subsequent protein identification.

### Protein Identification and Statistical Analysis

Protein identification and quantification were carried out using the ProteinPilot™ software v.4.0 (AB Sciex, Framingham, MA, United States). Each MS/MS spectrum was searched in the UniProt/SWISS-PORT database for *Arabidopsis thaliana* and in the UniProtKB/TrEMBL for *Viridiplantae* and *Brassica* genus (downloaded in 2014). Search parameters within ProteinPilot were set with trypsin cleavage specificity; methyl methanethiosulfonate (MMTS) modified cysteine as fixed modifications; biological modification “ID focus” settings, and a protein minimum confidence score of 95%. Thus, the identity of the protein from the analyzed peptide was confirmed, and the ratios of the peak areas of iTRAQ reporter ions were used to compare the relative abundance of the protein identified in each sample. Only proteins identified with at least 95% confidence, or a ProtScore (protein confidence measure) of at least 1.3 were reported (Fernández-Puente et al., 2011). Data were normalized for loading error by bias, and the background correction was calculated using the Pro Group™ algorithm (AB Sciex, Framingham, MA, United States). The results obtained from ProteinPilot™ were exported to Microsoft Excel for further analyses. The MS proteomic data have been deposited to the ProteomeXchange Consortium *via* the PRIDE partner repository with the dataset identifier PXD009097 and PXD009098.

After MS analysis, *p*-values for each replicate and identified protein were obtained. In order to carry out clearly statistical analysis, *p*-values from different replicates and same day were combined using Stouffer’s Z score, an established approach for combining information from multiple tests of the same null hypothesis (Pascovici et al., 2015). We considered statistically significant only those changes with a combined *p*-value ≤ 0.05 and an average ratio ≥1.2 (or ≤0.8). To perform the further analyses, we use the *A. thaliana* genes id. Finally, different tools, such as DAVID v. 6.8^1^ (Jiao et al., 2012) and MapMan v. 3.6.0RC1^2^ (Thimm et al., 2004), were used to perform Gene Ontology (GO) categories enrichment, pathway, and protein–protein interaction analyses.

## Results and Discussion

### Global Changes on the *Brassica oleracea* Proteome Infected by Xcc

To analyze *B. oleracea* proteomic response against the Xcc infection, proteomes from control and infected plants were compared combining iTRAQ technology and MS. To obtain proteomic information along the infection, we performed this analysis at 3 and 12 dpi. After the MS analysis, around 350 proteins were found by testing results against the specified databases. It is worth mentioning that we tried to identify pathogen proteins among the proteomic results by using several bacterial databases, but no proteins with bacterial origin was found in this study.

Among detected proteins, 26 and 58 proteins from 3 to 12 dpi samples, respectively, presented statistically significant differences between control and inoculated plants. Specifically, 14 proteins were increased, and 12 proteins decreased in inoculated versus control plants at 3 dpi (Table 1), whereas 35 proteins were increased and 23 decreased in inoculated versus control plants at 12 dpi (Table 2). Thus, inoculated plants showed serious damages at 12 dpi even in non-inoculated tissue, and the stronger proteomic changes were triggered at this infection point. This phenomenon could be explained since the doubled haploid line used in this work, “Early Big” is susceptible to Xcc race 1 attack. It is postulated that the main difference between resistant and susceptible plants is the timely recognition of the pathogen, and therefore, in defense activation. Although resistant plants are associated with the capability of a rapid and effective triggering of defense mechanisms, susceptible plants exhibit a slower response which is not able to avoid pathogen colonization and spread (Yang et al., 1997; O’Donnell et al., 2003).

**TABLE 1:** Proteins differentially induced 3 dpi. FC(I/C): the protein abundance ratio (inoculated/control);% cov: percentage of matching amino acids from identified peptides divided by the total number of amino acids in the sequence.

N	Protein name	Symbol	Uniprot code	ATG	*p*-value	Fold change (I/C)	% cov
	**GO response to bacterium**						
58*	Kunitz family trypsin and protease inhibitor protein	WSCP1R	Q8H0F0	AT1G72290	0.000	2.05	38.2
107	Kunitz trypsin inhibitor 1	KTI1	Q39369	AT1G73260	0.008	1.28	38.8
	**GO cellular protein modification process**						
70	Ubiquitin 7	RUB1/UBQ7	V5M3G8	AT2G35635	0.032	1.26	64.7
62	Related to ubiquitin 1	RUB2	P0C031	AT1G31340	0.032	1.26	64.8
	**GOs related with primary metabolism**						
1	Ribulose bisphosphate carboxylase large chain, chloroplastic	rbcL	A0A023VST5	ATCG00490	0.014	0.79	89.3
3	Ribulose bisphosphate carboxylase (small chain) family protein	RBCS1B	D6RRB9	AT5G38430	0.002	0.72	77.4
2*	Rubisco activase	RCA	P10896	AT2G39730	0.000	0.69	62.7
38	Alanine:glyoxylate aminotransferase	AGT1/SGAT	Q56YA5	AT2G13360	0.007	0.48	38.9
53*	Hydroxypyruvate reductase	HPR1	Q9C9W5	AT1G68010	0.012	0.34	34.7
11*	Glyceraldehyde-3-phosphate dehydrogenase C subunit 1	GAPC1	P25858	AT3G04120	0.000	0.75	61.8
135	Aldolase-type TIM barrel family protein	GOX2	M4EEX7	AT3G14415	0.007	0.75	34.3
18	ATP synthase subunit alpha, chloroplastic	atpA	P56757	ATCG00120	0.000	0.68	38.5
175	ATPase, V1 complex, subunit B protein	VAB2	Q9SZN1	AT4G38510	0.000	0.36	19.6
23	Photosystem II subunit O-2	PSBO2	Q9S841	AT3G50820	0.007	1.68	47.4
99	Glycine decarboxylase complex H	GDCH	M4E3B9	AT2G35370	0.044	1.24	47
	**Response to stimulus**						
3	Glyceraldehyde-3-phosphate dehydrogenase B subunit	GAPB	P25857	AT1G42970	0.008	0.67	67.8
4	Phosphoglycerate kinase 1	PGK1	Q9LD57	AT3G12780	0.001	0.68	68.6
	**Unclassified**						
75	Ribosomal protein L12-A	RPL12-A	M4E948	AT3G27830	1.5E-06	1.22	40.8
88	Ribosomal protein L3 family protein	BRA000285	M4C7V7	AT2G43030	0.001	1.23	39.9
	**• *Glucosinolates degradation/myrosinase***						
82*	GDSL-like lipase/acylhydrolase superfamily protein	N/A	Q39308	AT1G54020	0.004	1.31	29
	**• *Redox state***						
46*	hiGh cyclic electron flow 1	FBP/HCEF1	P25851	AT3G54050	0.011	0.75	29.7
96	Thioredoxin M-type 4	TRX-M4	M4EEN8	AT3G15360	0.013	1.31	43.8
	***• Auxins signaling***						
245	Coproporphyrinogen III oxidase	CPX1/LIN2	Q9LR75	AT1G03475	0.002	15.62	13
32	Chaperonin 20	CPN20	O65282	AT5G20720	0.016	1.82	79.8
	***• Others***						
58	Ribosome recycling factor, chloroplast precursor	RRF	Q9M1 × 0	AT3G63190	0.003	1.45	54.9
194	Winged-helix DNA-binding transcription factor family protein	BRA018301	M4DP61	AT2G30620	0.034	1.27	52.5

GO, Gene Ontology. * Shared proteins between dpi.Proteins were listed in descending order according their score, lower N number means higher identification score.

**TABLE 2:** Proteins differentially induced 12 dpi. FC(I/C): the protein abundance ratio (inoculated/control);% coverage: percentage of matching amino acids from identified peptides divided by the total number of amino acids in the sequence.

N	Protein name	Symbol	Uniprot code	ATG	p-value	Fold change (I/C)	% cov
	**Primary metabolism**						
5	Transketolase	TKL-1	Q8RWV0	AT3G60750	0.001	2.12	54.3
11*	Hydroxypyruvate reductase	HPR1	M4CJ91	AT1G68010	0.004	1.68	65.5
1	Fructose-bisphosphate aldolase 2	FBA2	Q944G9	AT4G38970	0.015	1.66	62.3
20	Carbonic anhydrase 1	CA1	M4FDE2	AT3G01500	0.000	1.36	63.5
444	MA3 domain-containing protein	-	V4KTB4	AT1G22730	0.007	1.27	18
206	Phosphoglycerate/bisphosphoglycerate mutase	PGM	F2E7L1	AT1G78050	0.005	1.26	21.1
411	Photosystem II family protein	PSB27	R0GS55	AT1G03600	0.042	1.26	16.8
418*	Glyceraldehyde-3-phosphate dehydrogenase C subunit 1	GAPC1	D0R8V8	AT3G04120	0.009	1.21	36
21	ATP synthase subunit beta	atpB	P19366	ATCG00480	0.000	1.18	53.8
56	Protein containing PDZ domain, a K-box domain, and a TPR region	ZKT	M4D610	AT1G55480	0.017	0.72	46.3
32	Sedoheptulose-bisphosphatase	SBPASE	P46283	AT3G55800	0.000	0.71	52.2
33	Chaperonin 60 beta	CPN60B	M4EPY7	AT1G55490	0.000	0.55	40.6
2	Photosystem II subunit P-1	PSBP1	Q42029	AT1G06680	0.000	0.48	55.5
18*	Rubisco activase	RCA	X2C5E1	AT2G39730	0.000	0.23	52.8
19	Photosystem II subunit Q-2	PSBQ2	M4EL96	AT4G05180	1.48E-10	0.15	75.2
	**Biotic stress processes**						
	**• *Signaling***						
66	Plasma-membrane associated cation-binding protein 1	PCAP1	X4Z1A4	AT4G20260	0.003	1.54	70.1
89	General regulatory factor 2	GRF2	Q01525	AT1G78300	0.012	1.67	54.1
	**• *Defense genes***						
37 *	Kunitz family trypsin and protease inhibitor protein	WSCP1	Q7GDB3	AT1G72290	0.000	0.54	67.4
85	Pathogenesis-related protein 1	Ypr1/PR1	Q96344	AT2G09750	0.012	2.29	45.1
	**• *Redox state***						
30*	High cyclic electron flow 1	FBP/HCEF1	Q9ZP29	AT3G54050	0.033	0.74	53.2
146	Thioredoxin superfamily protein	-	A0A067KWB6	AT3G11630	0.017	1.27	49.3
26	Thioredoxin superfamily protein	PRXQ	M4F5K9	AT3G26060	0.000	0.77	70.8
369	Thioredoxin F-type 1	TRXF1	M4DY21	AT3G02730	0.021	0.80	25.8
314	Pentapeptide repeat-containing protein	BRA026982	M4EDX2	AT1G12250	0.037	0.76	19.7
298	Cell wall integrity/stress response component	BRA031804	M4ESM4	AT5G66090	0.020	0.79	21.6
31	Fe superoxide dismutase 1	SODB	U3N2Z5	AT4G25100	0.000	0.64	54.3
	***• Proteolysis***						
112	LTP2, PR-14 like protein	LTP2	Q42589	AT2G38530	0.024	0.50	37.3
169	LTP3, PR-14 like protein	LTP3	Q9ZSL7	AT5G59320	0.001	0.24	45.5
194	Hypothetical protein	N/A	M4D722	AT1G21500	0.001	0.71	47.1
388	Ubiquitin-conjugating enzyme 36	UBC36	R7W7E7	AT1G16890	0.006	1.26	10.4
238	Ferredoxin-NADP[+]-oxidoreductase 2	FNR2	M4D6U3	AT1G20020	0.023	1.27	67
83	CLPC homolog 1	CLPC1	M4E1V1	AT5G50920	0.034	1.22	38.2
	**• *Glucosinolates degradation/myrosinase***						
32*	GDSL-like lipase/acylhydrolase superfamily protein	BnaC06g06760D	P93064	AT1G54020	0.000	1.32	51.2
	**• *Phenylpropanoids-lignin biosynthesis***						
430	S-adenosyl-L-methionine-dependent methyltransferases	CCoAOMT1	V4MBF9	AT4G34050	0.003	1.21	20.2
	**• *Hormone signaling***						
188	CP12 domain-containing protein 1 (BRA)	CP12-1	M4CJS5	AT2G47400	0.007	0.71	32.3
178	CP12 domain-containing protein 2 (BRA)	CP12-2	M4CH16	AT3G62410	0.013	0.51	19.4
323	Aldolase-type TIM barrel family protein (AUX)	RSR4	I0Z028	AT5G01410	0.029	1.28	25.1
84	Mannose-binding lectin superfamily protein (JA)	JR1	M4E064	AT3G16470	0.018	1.34	50.7
90	Dehydrin family protein (GA)	ERD14	M4DH02	AT1G76180	0.014	1.38	56.4
219	Xyloglucan endotransglucosylase/hydrolase 24 (GA)	XTH24	R0GXB3	AT4G30270	0.036	1.26	17
148	Uridylyltransferase-like protein (CK)	ACR11	M4EB60	AT1G16880	0.006	0.77	36
29	Glutamine synthetase 2 (CK)	GS2	M4F2U3	AT5G35630	0.000	2.11	49.8
27	Peroxisomal NAD-malate dehydrogenase 2 (CK)	PMDH2	M4EIK9	AT5G09660	0.001	1.29	54.8
	**Unclassified**						
81	Ribosomal protein L12-C	RPL12-C	Q8LBJ7	AT3G27850	0.002	0.60	43.3
191	Ribosomal protein S5/elongation factor G/III/V family protein	LOS1	M4DL98	AT1G56070	0.016	1.58	26.5
58	UbiA prenyltransferase family protein	G4	M4CGE0	AT3G51820	0.000	2.59	49
6	Cobalamin-independent synthase family protein	MS1	O50008	AT5G17920	0.002	1.61	46.8
408	VIRB2-interacting protein 2	BTI2	B9I788	AT4G11220	0.017	1.52	9.8
76	Cyclophilin 38	CYP38	M4FDE4	AT3G01480	0.021	1.27	35.8
304	beta-1,3-glucanase 2	BGL2	Q2VT22	AT3G57260	0.027	1.26	19.3
95	ADP glucose pyrophosphorylase large subunit 1	APL1	M4CDE0	AT5G19220	0.011	1.23	34.5
325	Chloroplast beta-amylase	CT-BMY	M4EBS1	AT4G17090	0.003	1.22	16.6
198	Zinc-binding dehydrogenase family protein	BRA006381	M4CQ92	AT5G16990	0.015	1.21	19.2
7	glutamate synthase 1	GLU1	Q9ZNZ7	AT5G04140	0.021	1.14	33.1
13	Oxidoreductase, zinc-binding dehydrogenase family protein	AOR	Q9ZUC1	AT1G23740	0.000	0.79	68.1
126	Tetratricopeptide repeat-like superfamily protein	TPR	M4CKY9	AT2G44920	0.006	0.70	58.6
324	-	Sb06g016740	C5YGH3	-	0.011	0.76	10.5
21	-	atpB	P19366	ATCG00480	0.000	1.18	53.8

*Shared proteins between dpi. Proteins were listed in descending order according their score, lower N number means higher identification score.

After the recognition of the pathogen, the responses observed involved important signaling proteins from different cell processes, from primary metabolism, such as photosynthesis or photorespiration, to other complex processes such as redox homeostasis, hormone signaling, or defense mechanisms (Table 1). Moreover, the comparison of the 3 and 12 dpi results showed that only 6 proteins were shared between dpi. Among them, three proteins showed the same behavior at the two times analyzed.

### Early Proteomic Response

Plants facing bacterial infection presented perturbations in multiple pathways from essential mechanisms to more unknown and complex pathways. The GO enrichment analysis revealed that the differentially present proteins were involved on different biological processes. In the case of the decreased proteins, they were enriched in plant growth and primary metabolism, such as “photorespiration” (*p*-value = 2.5 × 10^–4^), “oxidative photosynthetic carbon pathway” (*p*-value = 4.4 × 10^–3^), “gluconeogenesis” (*p*-value = 6.8 × 10^–3^), or “glycolytic process” (*p*-value = 5.1 × 10^–4^). Particularly, two of the central photosynthetic proteins, ribulose bisphosphate carboxylase (rubisco) and rubisco activase were repressed by the Xcc infection. This means a reorientation of resources from cellular maintenance, growth, and reproduction toward defense and signaling pathways. Similar observations have been reported previously in other works using different plant–pathogen interaction models (Mitra and Baldwin, 2008; Bilgin et al., 2010). A downregulation of proteins related to photosynthesis, energy, and defense was also observed in previous studies of *B. oleracea*–Xcc infection (Villeth et al., 2016) and rapeseed (*B. napus*–Xcc infection) by Islam et al. (2021). Most of the proteins decreased in the earlier response were involved in energetic metabolism, whereas the later response was characterized by a recovery of primary metabolism. We show that the regulation of photosynthesis seems to be essential for a response against Xcc, even at an early stage of infection. A similar result was reported by Ribeiro et al. (2018) at an early stage of infection (24 h after the Xcc inoculation of *B. oleracea*).

Regarding 14 induced proteins, only four of them could be categorized into two GO terms, “response to bacterium” (*p*-value = 1.7 × 10^–2^) and “cellular protein modification process” (*p*-value = 2.6 × 10^–2^). The proteins classified into the former GO term, KTI1 and WSCP1R, are kunitz trypsin inhibitors 1, which belong to the so-called pathogenesis-related proteins (PRs) type 6. PRs are apoplastic and ubiquitous proteins present in the plant kingdom. Particularly, type 6 PRs, a subclass of serine proteinase inhibitors, have the property to bind proteinases and control their activity, and therefore could have multiple roles in plants (Sudisha et al., 2012). Regarding their role in plant defense, they are also able to interact with proteinases from plant-attacking organisms. However, while their effectiveness against fungi, virus, or insects have been proven, their activity on microbial proteinases has not been studied intensively yet (Sels et al., 2008).

In the second GO term, “cellular protein modification process” was classified into two post-translational protein modifiers, RUB1 and RUB2, implicated in the ubiquitination pathway and therefore, in protein degradation. The ubiquitin pathway is necessary to tag proteins that should be degraded. Ribeiro et al. (2018) found that in a highly resistant plant to Xcc proteins related to ubiquitination showed reduced abundance at 24 h after inoculations, which may indicate a negative regulation of this pathway, In contrast, among unclassified increased proteins, we found two essential structural constituents of ribosomes. These results suggest that a complex process of protein synthesis–degradation is activated in the first stage of the Xcc infection. It is well known that most of plant molecular processes are regulated by a balanced synthesis and degradation of proteins that control them. In healthy plants, proteolysis is a process commonly associated with plant senescence, essential for mobilization of nutrients from old tissues to growing or sink organs, or in other words, to nutrient reallocation (Diaz-Mendoza et al., 2016). However, increasing evidence has been found in the past decade for the role of proteolysis in plant defense. Different studies indicated that ubiquitin machinery could act as negative or positive regulators of defense response depending on their substrates, although the insights of its role remain to be determined (Delauré et al., 2008; Santos et al., 2019). Thus, not surprising that several works reported that pathogens have developed tactics to influence on susceptible host’s ubiquitin proteasome system in order to promote their own survival (Dreher and Callis, 2007). The bacterial effectors could be degraded by proteasomes; therefore, they can interfere in the system, act as an ubiquitin ligase, or inhibit the specific ubiquitin–proteasome system (UPS) steps (Santos et al., 2019). Given this scenario, proteolysis machinery could have been modified because of the Xcc infection.

Finally, we found that the protein coproporphyrinogen III oxidase strongly increased after the Xcc inoculation. This protein is a key enzyme of the tetrapyrrole biosynthesis pathway. This protein mediates disease resistance in plants by the salicylic acid pathway. According to Guo et al. (2013), this protein modulates apoptosis in *A. thaliana* to powdery mildew disease. Apoptotic changes in vascular tissues are known to promote pathogen establishment, which could explain its increase only during the first stage of the Xcc infection (Gupta et al., 2013).

### Late Proteomic Response

**FIGURE 2:**
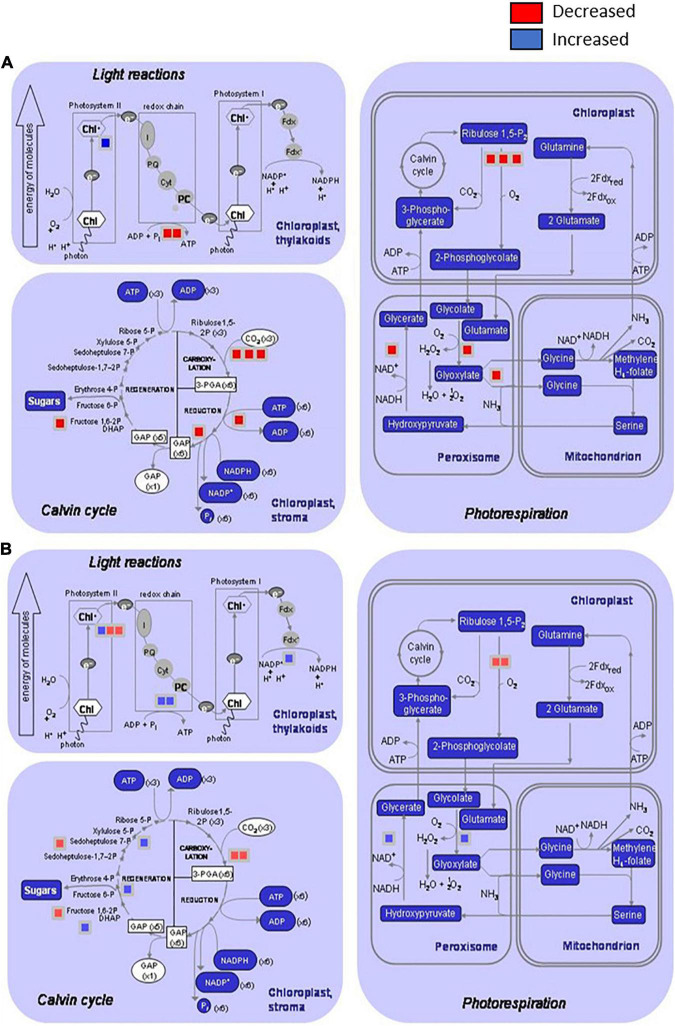
Distribution of the differentially induced proteins involved on primary metabolism processes after infection of *Brassica oleracea* leaves by *Xanthomonas campestris* pv. *campestris*. Graphics show proteins decreased or increased in three processes: light reactions (photosystem I, Photosystem II, and redox chain), Calvin cycle, and photorespiration (in chloroplast, peroxisomes, and mitochondrions) by using the MapMan software. **(A)** 3 dpi and **(B)** 12 dpi. Red square: downregulated protein; blue square: upregulated protein. At 3 dpi, two proteins involved in light reactions, six proteins in Calvin cycle, and six proteins in photorespiration were significantly reduced in infested plants versus control plants. Only one protein involved in Photosystem II was significantly increased at this stage. At 12 dpi, two proteins involved in Photosystem I, four proteins related to Calvin cycle, and two proteins involved photorespiration in chloroplasts were significantly reduced in infested plants versus control plants. Protein induction was stronger at 12 dpi than 3 dpi, and four proteins related to light reactions, three involved in Calvin cycle and two proteins involved in photorespiration at peroxisome level were differentially increased.

As we introduced in previous sections, the global results indicated a more powerful response in the case of 12 dpi, since the regulation of more proteins was altered in comparison with the earlier response (Figure 2). However, several of the altered pathways were shared between the two times analyzed, such as processes related to primary metabolism.

Interestingly, some of the primary metabolism pathways that were diminished during the early response presented a normal behavior at 12 dpi, or even an overactivation, in comparison with control plants. This behavior is illustrated in Figure 2, which shows the distribution of the differentially regulated proteins at 3 and 12 dpi involved in primary metabolism processes. At 3 dpi, different steps of pathways related to light reactions, Calvin cycle, and photorespiration in both chloroplasts and peroxisomes were diminished. In contrast, other steps of the mentioned processes were boosted at 12 dpi. So, in the first stages of the disease plant, metabolism was redirected toward signaling and defense mechanisms at the expense of energetic metabolism, and during the late response, some features from primary metabolism were recovered or even enhanced. Villeth et al. (2016) postulated that the resistance is correlated with the ability of the plant to keep sufficient photosynthesis activity, and therefore to have the necessary energy to trigger defense mechanisms. Furthermore, considering that “Early Big” is a susceptible line, primary metabolism recovering could be delayed in comparison with a resistant line, being too late for the plant survival. So, this result reinforces the important role of photosynthesis during pathogenesis and supports the idea about the main difference between resistant and susceptible plants that could be based on the timely response activation, including basal metabolism maintenance.

The overall defense level of a plant is a function of traits related to primary metabolism and the concentrations of defensive secondary metabolites. According to the defense trade-off hypothesis, plants possess a limited pool of resources that can be invested either in growth or in defense (Huot et al., 2014; Zust and Agrawal, 2017). For a long time, it was thought that the reason for the growth–defense trade-off might be a question of energy resources. Recently, Neuser et al. (2019) establish that the actual underlying reason is the incompatibility of the molecular pathways regulating plant growth and defense. In this study, plants at 12 dpi showed an increase in proteins involved both in growth and in biotic stress processes. Proteins related to the primary metabolism were recovered during the late response, possibly because the response is being evaluated in a susceptible material.

**FIGURE 3:**
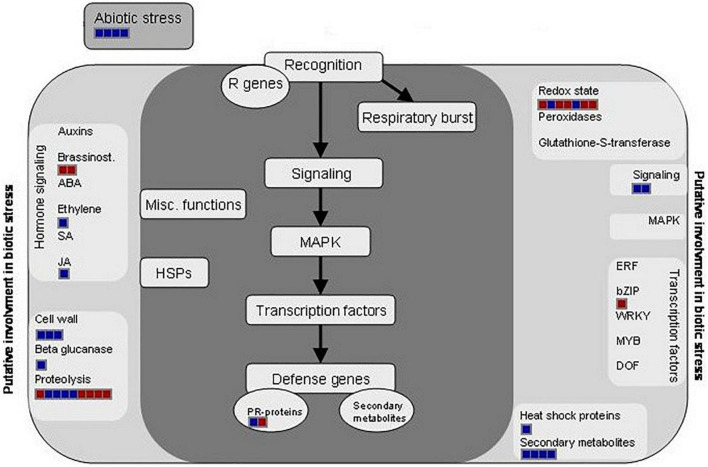
Distribution of the differentially regulated proteins at 12 dpi and involved in biotic stress processes (hormone signaling, secondary metabolism activation, and redox homeostasis) by using the MapMan software and after the infection of *Brassica oleracea* leaves by *Xanthomonas campestris* pv. *campestris*. Red square: downregulated protein; blue square: upregulated protein.

Besides this, other processes were modified during a well-stablished Xcc infection. Among them, some are involved in biotic stress mechanisms (Figure 3 and Table 2), such as hormone signaling, secondary metabolism activation, or redox homeostasis. At this stage, the results indicate that there was an accumulation of proteins involved in oxidative stress and proteolysis-related protein induction. As occurred during the earlier response, our results indicated a complex regulation of proteolysis. Interestingly, among the proteins classified in “proteolysis” process, we found the lipid transfer protein 2 (LTP2) and the lipid transfer protein 3 (LTP3). LTPs are defense proteins that are components of plant innate immunity and exhibit a broad spectrum of biological activities. LTPs were named because of their ability to transfer various types of lipids between membranes; however, the gene expression of some LTPs was also found to respond to pathogen infection, so they were also classified as PR-14 proteins (Sels et al., 2008). Despite the details of LTP behavior remain unclear, it seems that these proteins have a role during adaptation to biotic stress factors (Jung et al., 2003). Different works demonstrated LTP capability to inhibit the fungal growth *in vitro* (Safi et al., 2015), and Julke and Ludwig-Muller (2016) showed that an overexpression of different LTP genes led to reduced clubroot susceptibility, a disease of Brassicaceae caused by *Plasmodiophora brassicae*. Surprisingly, our result showed a strong decrease of LTP2 and LTP3 at 12 dpi. The genetic complexity of LTPs suggests that a specific member of the gene family may be involved in a particular biological function, which could explain the observed decrease. Different works relate LTP2 (Jacq et al., 2017) and LTP3 (Pagnussat et al., 2015) with basal processes, such as plant growth and reproduction, fact that goes in agreement with the other results obtained in this work.

It is well known that upon the recognition of pathogen infection, plants trigger the so-called oxidative burst, a rapid production of reactive oxygen species (ROS) in the apoplast to cope with the invader (Torres, 2010). This defense mechanism is usually classified as an early plant immune response and is directly related to the hypersensitive response (HR). However, the maintenance of ROS homeostasis is crucial due to the continuous exposure to high levels of ROS also led to molecular damages in the plant, such as protein denaturation or DNA base oxidation (Belozerskaya and Gessler, 2007). Different proteomic studies showed an induction of ROS-scavenging proteins, such as thioredoxins, superoxide dismutases, and glutaredoxin-like proteins, after invasion by different plant pathogens in a timely manner (Kim et al., 2013; Li et al., 2016). In this work, the abundance of several proteins related to the ROS scavenging was modified after the Xcc infection (Figure 3). Nevertheless, unlike the works mentioned, most of them were decreased at 12 dpi. Despites this result goes in agreement with the level of damages in the whole plant, it is not an expected response after so many days of infection. Similar results were found by Kang and Udvardi (2012). They investigated the expression of ROS-scavenging genes in alfalfa under drought conditions. They observed a strong increase of some ROS-scavenging genes under optimal conditions and a decrease of others, such as thioredoxins, under drought stress. Thus, they concluded that the ROS scavenging is very complex process, and it cannot be assumed that it is always beneficial to increase the expression of ROS-scavenging genes during stress, as reported in earlier studies. Proteins related to the hormone signaling were either reduced (brassinosteroids) or increased [ethylene, jasmonic acid (JA), and salicylic acid (SA)] at 12 dpi. It is well known that phytohormones, ethylene and jasmonate, play a major role in defense responses against pathogens and herbivorous pests. Parallel approaches have demonstrated that SA is required for the activation of both pathogen-associated molecular patterns (PAMPs)-triggered immunity (PTI) and effector-triggered immunity (ETI) (Figure 3). Plant PRs were also involved at this stage, and PR1 showed the highest abundance ratio (inoculated/control), as it was previously reported (Table 2).

In conclusion, the current study represents an extensive analysis of the protein profile of *B. oleracea* in response to the Xcc inoculation in susceptible interactions. Stronger proteomic changes associated with the susceptible *B. oleracea*–Xcc interaction were obtained at 12 dpi in comparison with 3 days. This study underscores the importance of an efficient balance between the basal metabolism and other processes, such as signaling or defense mechanisms, during the *B. oleracea*–Xcc interaction and suggests that this could be the key difference between a susceptible and resistant plant. In addition, other processes, such as proteolysis or ROS scavenging, play a remarkable role during pathogenesis. Further availability of genotypes with resistance to Xcc allow us to deeply study the processes highlighted here and determine which proteins contribute to the Xcc pathogenesis response. The approach used in this study may be especially useful in further analyses in order to understand plant’s response mechanisms against this important plant pathogen. Our results provide new insights into molecular mechanisms that may be involved in responses of *B. oleracea* to Xcc.

## Data Availability Statement

The original contributions presented in the study are publicly available. This data can be found here: “The mass spectrometry proteomics data have been deposited to the ProteomeXchange Consortium via the PRIDE partner repository with the dataset identifiers PXD009097 and PXD009098.”

## Author Contributions

MEC and PV conceived the project and designed the experiments. MT, PV, VR, and MEC assisted with the setup of plant experiments and conducted laboratory work. MT analyzed the data and wrote the manuscript. MEC supervised the manuscript. All authors read and approved the final manuscript.

## Conflict of Interest

The authors declare that the research was conducted in the absence of any commercial or financial relationships that could be construed as a potential conflict of interest.

## Publisher’s Note

All claims expressed in this article are solely those of the authors and do not necessarily represent those of their affiliated organizations, or those of the publisher, the editors and the reviewers. Any product that may be evaluated in this article, or claim that may be made by its manufacturer, is not guaranteed or endorsed by the publisher.

## Footnotes

1
https://david.ncifcrf.gov/

2
https://mapman.gabipd.org/mapman
